# Sailors and Anti-Scorbutics

**Published:** 1888-05-19

**Authors:** 


### SAILORS AND ANTI-SCORBUTICS.

THE question has been raised in Parliament as to whether the masters of British ships (whatever may be meant by the word British) are compelled to serve out lime, lemon-juice, or other anti-scorbutic to crews, when such ships are not coming to or going from the United Kingdom. The Merchant Shipping Acts are the worst drawn measures in the Statute-book, and they are made more confounding by the annual muddles that some people call Amendment Acts. The Board of Trade has no discretion vested in its staff to interpret the laws. They are the executive or administrative department; and if any dispute arises as to the proper construction to be placed on any clause, the Courts are open to protesters. It is said that the law as laid down in the Merchant Shipping Act, 1867, is impliedly restricted to vessels bound from or to the United Kingdom. This method of constructing the law is as bad as leaving the whole affair to imagination. Implication is not a satisfactory term to rely upon. We shall make an effort to explain the law, and then leave the critics to draw what conclusions they please from our exposition. Section 1 of the Act of 1867 provides that "this Act shall be construed with and as part of the Merchant Shipping Act, 1854." Clause 4 enacts that the rules in paragraphs 1 to 6 shall be observed with respect to medicines, medical stores, and anti-scorbutics. The Board of Trade (par. 1) shall publish scales of medicines and medical stores, and the owners of every ship navigating between the United Kingdom and any place out of the same shall cause a supply of medicines and medical stores to be taken on board their vessels. We then reach par. 3, which says that no lime or lemon juice shall be deemed fit for use of crew or passengers unless fortified and bonded in the manner prescribed. The master or owner (par. 4) of every such foreign-going ship, except those bound to European ports, or Mediterranean, or East Coast of America north of 35 deg. latitude, shall keep on board a sufficient quantity of lime or lemon juice, or other anti-scorbutics composed of such materials as may be approved by her Majesty. Then follows the mandate which has to be acted upon reading thus: "The master of every foreign-going ship shall serve out the lime or lemon juice with sugar, or other anti-scorbutic, to the crew so soon as they have been at sea for ten days, and during the remainder of the voyage, except during such time as they are in harbour, and are there supplied with fresh provisions." Paragraphs 1 and 2 are devoted to medicines and medical stores, and expressly confine the operation of the statute to ships navigating between the United Kingdom and any place beyond Paragraphs 3, 4, and 5, refer to lime, lemon juice, or other anti-scorbutics, and nothing further is said than that the compulsory requisition is limited to foreign-going ships. As the second paragraph speaks of ships navigating between the United Kingdom and any place out of it, the inference is (so say officials) that the entire section should be read as one whole, and in such a case the Act of 1867 does not apply to owners or masters whose ships are not sailing between ports of the United Kingdom "and any place out of the same." A special pleader would show that this proviso is restricted to "any place out of the same," and does not embrace to the same. There is much that might be urged on this point; but we skip over this little bit of slovenly composition to say that section 224 of the Merchant Shipping Act was repealed and embodied almost verbatim in clause 4 of the Merchant Shipping Act, 1867, extracts from which we have given above. Section 224 was in part 3 of the Act of 1854, and though section 4 of the Act of 1867 is in reality a substitute, no statement is made that this order should be followed. In the Merchant Shipping Act, 1862, the separate parts of chapters were delimited, and from that mode of concoction the reader is enabled to gain instruction. To part 3 of the Act of 1854 there is an introductory and interpretation clause, which provides that so much of the third part as relates to the provisions, health, and accommodation of seamen shall apply to all ships registered in her Majesty's dominions abroad. This is wholesale legislation with a vengeance. It might have been acceptable previous to 1854, when the colonists could not make their own laws; but in our day an Imperial Parliament sitting at Melbourne has as much power or right to pass laws as the Imperial Parliament assembled in London. If that interpretation clause is applied literally to the Act of 1867, all the Empress of India's ships and vessels belonging to the colonies would have to be supplied with lime or lemon juice if they left for a foreign port. Another paragraph in section 109 of the Act of 1854 provides that the whole of part 3 shall apply to all sea-going ships registered in the United Kingdom, and also to all ships registered in any British possession, and employed in trading or going between any place in the United Kingdom and any place not situated in the possession to which the vessels belong. We might read this by importing words of our own—"where not otherwise excepted." Those words are not in the Act; but almost identical language is used, namely—"unless the context or subject-matter requires a different application;" and it then goes on to give the express definitions we have instanced. If this is not a perfect mess, we know not where such a hash may be found. After a careful consideration of the Acts of Parliament we are forced to conclude that the Act does not seem to apply to vessels leaving the United Kingdom with no intention of returning; but whether it applies to foreign-going ships, British and Colonial, when plying abroad, is a speculative question which we defy anyone to reconcile to our satisfaction with the conflicting statutes.—*Liverpool Journal of Commerce.*

